# Primary Causes of Dental Caries

**Published:** 1893-09

**Authors:** Gustavus North

**Affiliations:** Springville, Iowa


### Primary Cause of Dental Caries.

BY GUSTAVUS NORTH, A.M., D.D.S., SPRINGVILLE, IOWA.

The cause of dental caries is a subject that always brings forth considerable discussion. In this short paper only one of the primary causes of decay will be mentioned, namely, that of lactic fermentation, from foreign substances, food, etc., that find lodgment in and around the teeth and gums.

The lactic acid ferment, coming in contact with this foreign substance soon sets up lactic fermentation which results in lactic or butyric acids, according to the character of the foreign substance and the extent of the fermentation, the same ferment producing both the acetic fermentation and the butyric fermentation, the latter being the advance stage of the former.

The liberation of these acids in their nascent state induces action upon the structure of the teeth and gives rise to conditions favoring necrosis.

Teeth that are normal and well developed are not easily acted upon by the acetic fermentation. But malformed teeth, either from heredity, eruptive diseases, or any other cause that may stunt or check development of the child, from the first to the ninth year of age, during the period of formation and development of the enamel of the permanent teeth, produce a condition favorable to the acetic fermentation in the foreign substance lodged around the teeth.

The fermentation taking place in contact with defective teeth produces a good soil for the micro-organisms of caries to grow and develop in.

Caries is therefore a chemico-parasitical process, consisting of two distinctly marked stages: first, the chemical action of the fermented acids softening the inorganic matter of the teeth, second, that of dissolution of the softened dentine by bacteria.

